# Correction: Pujic et al. The Proteogenome of Symbiotic *Frankia alni* in *Alnus glutinosa* Nodules. *Microorganisms* 2022, *10*, 651

**DOI:** 10.3390/microorganisms10112229

**Published:** 2022-11-11

**Authors:** Petar Pujic, Nicole Alloisio, Guylaine Miotello, Jean Armengaud, Danis Abrouk, Pascale Fournier, Philippe Normand

**Affiliations:** 1 Ecologie Microbienne, CNRS, UMR5557, Université Lyon 1, Université de Lyon; INRA, UMR1418, 7330 Villeurbanne, France; 2 Département Médicaments et Technologies pour la Santé (DMTS), CEA, INRAE, Université Paris-Saclay, SPI, 30200 Bagnols sur Cèze, France

The authors wish to make the following corrections to this paper [1].

## Text Correction

The original version of this article contained an error on the control of the proteogenomic experiment, which was not nitrogen-fixing BAP—grown culture (without NH_4_^+^), but a nitrogen-replete BAP+ (containing 5 mM NH_4_^+^) culture. We corrected this error by replacing “N-fixing” by “N-replete” throughout the text.

Examples of corrections are given below:

In the **Abstract**:

A proteogenomic analysis of symbiotic *Frankia alni* was done by comparing those proteins more and less abundant in *Alnus glutinosa* nodules relative to N-replete pure cultures with propionate as the carbon source and ammonium as the nitrogen-source. There were 250 proteins that were significantly overabundant in nodules at a fold change (FC) ≥ 2 threshold, and 1429 with the same characteristics in in vitro nitrogen-replete pure culture.

In the **Materials and Methods**:

As a reference, *F. alni* cells were inoculated after syringing with a series of needles (21G, 23G, 25G, 27G) and grown for 10 days (corresponding to the end of the exponential phase) in 250 mL of BAP medium with ammonium (5 mM) in agitated 500 mL Erlenmeyer flasks [25] buffered to pH 6.5. No vesicles could be found.

As here in the **Results**:

The three biological replicates of symbiotic *Frankia alni* overproduced at a fold change of ≥2250 proteins (Supplementary Table S1) using a nitrogen-replete propionate-fed pure culture as reference, of which 100 had an FC ≥ 4.38 (Table 1).

And here:

Among *F. alni* proteins, the nitrogenase proteins were the most overabundant with 7 among the 10 highest using as reference a nitrogen-replete pure culture.

As here in the **Legend to Figure 1**: 

Figure 1. Circular map of the genome of *Frankia alni* with the proteins over-abundant in nodules relative to a nitrogen-replete pure-culture (FC ≥ 2) positioned along the genome.

As here in the **Title** of Supplementary Materials Table S1:

Table S1: List of *Frankia* proteins identified in nodules and in a nitrogen-replete pure culture and their spectral counts.

And as here in the **Acknowledgments**: 

Thanks are expressed to Elise Lacroix for Greenhouse management (Université de Lyon) and to Aude Herrera-Belaroussi (Université de Lyon) for the nitrogen-replete *Frankia* cells.

## Reference

Furthermore, reference Clavijo et al. (2015) had been truncated, it should be as:

11 Clavijo, F.; Diedhiou, I.; Vaissayre, V.; Brottier, L.; Acolatse, J.; Moukouanga, D.; Crabos, A.; Auguy, F.; Franche, C.; Gherbi, H.; et al. The *Casuarina* NIN gene is transcriptionally activated throughout *Frankia* root infection as well as in response to bacterial diffusible signals. *New Phytol*. **2015**, *208*, 887–903.

The authors state that the scientific conclusions are unaffected. This correction was approved by the Academic Editor. The original publication has also been updated.
